# Evaluation of Starch–Garlic Husk Polymeric Composites through Mechanical, Thermal, and Thermo-Mechanical Tests

**DOI:** 10.3390/polym16020289

**Published:** 2024-01-20

**Authors:** Cynthia Graciela Flores-Hernández, Juventino López-Barroso, Beatriz Adriana Salazar-Cruz, Verónica Saucedo-Rivalcoba, Armando Almendarez-Camarillo, José Luis Rivera-Armenta

**Affiliations:** 1 Departamento de Metal Mecánica—División de Estudios de Posgrado e Investigación, Instituto Tecnológico de Querétaro/Tecnológico Nacional de México, Av. Tecnológico S/n Esq. Gral. Mariano Escobedo, Santiago de Querétaro 76000, Querétaro, Mexico; cynthiagraciela84@gmail.com (C.G.F.-H.); juventino.lb@queretaro.tecnm.mx (J.L.-B.); 2 Centro de Investigación en Petroquímica, Instituto Tecnológico de Ciudad Madero/Tecnológico Nacional de México, Pról. Bahía de Aldair y Ave. de las Bahías, Parque de la Pequeña y Mediana Industria, Altamira 89603, Tamaulipas, Mexico; beatriz.sc@cdmadero.tecnm.mx; 3 Ingeniería en Procesos Biotecnológicos y Alimentarios, Instituto Tecnológico Superior de Tierra Blanca/Tecnológico Nacional de México, Av. Veracruz s/n Esquina Héroes de Puebla, Col. Pemex, Tierra Blanca 95180, Veracruz, Mexico; veronica.saucedo@itstb.edu.mx; 4 Departamento de Ingeniería Química, Tecnológico Nacional de México/Instituto Tecnológico de Celaya, Antonio García Cubas Pte. #600 Esq. Av. Tecnológico, Celaya 38010, Guanajuato, Mexico; armando@iqcelaya.itc.mx

**Keywords:** starch, garlic husk, evaporation casting, composites, biopolymers

## Abstract

The present work evaluates the influence of different properties of composite materials from natural sources. Films were prepared using the evaporative casting technique from corn starch reinforced with a waste material such as garlic husk (GH), using glycerin as a plasticizer. The results of the syntheses carried out demonstrated the synergy between these materials. In the morphological analysis, the compatibility and adequate dispersion of the reinforcer in the matrix were confirmed. Using Fourier transform infrared spectroscopy (FTIR), the interaction and formation of bonds between the matrix and the reinforcer were confirmed by the presence of some signals such as S-S and C-S. Similarly, thermogravimetric analysis (TGA) revealed that even at low concentrations, GH can slightly increase the decomposition temperature. Finally, from the results of dynamic mechanical analysis (DMA), it was possible to identify that the storage modulus increases significantly, up to 115%, compared to pure starch, especially at low concentrations of the reinforcer.

## 1. Introduction

The planet increasingly faces environmental problems due to the indiscriminate use of plastics and raw materials derived from petrochemical sources, and these materials are being used in many sectors of the world economy [1]. The demand and exploitation of polymers are due to their low production cost, lightness, versatility, and durability (some of which remain in the environment for hundreds of years) [2]. However, with short periods of use, they quickly become waste. The accumulation of waste and its disposal produce a greater ecological footprint and a severe environmental problem with accumulation in landfills [2]. Additionally, plastic waste represents a threat to marine life and other wildlife, public health, and the economic, social, and environmental sectors that directly depend on the health of marine life [3,4]. The problem of waste disposal, which is based on a linear economy, has worsened over the years due to the greater use of disposable, durable materials resistant to biodegradation [1,5]. Unfortunately, the current transition rate from petrochemical polymers to biodegradable polymers is low and inconsistent. For this reason, the socioeconomic system must be based on a circular economy and the use of biodegradable materials to mitigate environmental pollution derived from plastics [2]. On the other hand, waste recycling is considered one of the main processes to significantly minimize the consumption of virgin materials and increase the amount of recycled waste [6]. Furthermore, using biodegradable and ecological materials is another alternative to mitigate environmental pollution [7]. Developing new materials based on renewable and sustainable sources to prepare new packaging, bags, and other products in the industrial sector and in different fields of materials science would align with the concepts of a circular economy promoted by different international policies [8,9]. 

A few researchers have focused on the development of plastic materials that are friendly to the environment and have searched for combinations of natural polymers, including proteins, polysaccharides, and synthetic polymers from both chemical and biological sources, as well as microorganism syntheses [10,11,12,13,14]. Biopolymers represent a group of materials that are formed from natural sources, which implies that they can be disposed without significant damage to the nature; therefore, these kinds of materials represent a good option for applications with a short lifespan. Starch is an important renewable biopolymer due to its low cost, abundance in nature, and biological and biodegradable base, which could meet the conditions for minimizing the impact of plastics [15,16,17]. For example, 75% of this polymer are used to manufacture containers and packaging [18]. Recently, several application areas have been reported such as green chemistry, water treatments, medical applications, and electronic industry [19]. However, it has disadvantages, such as poor mechanical and barrier properties associated with its high affinity for water due to its hydrophilic character [20,21]. The evaporative casting technique consists of dissolving a polymer in appropriate solvent and subsequent evaporation to obtain a film. A solution to improve the starch film properties could be to use different strategies to improve the properties of starch polymers, such as the incorporation of particles, nanoparticles, and fibers, thus developing a biocomposite a mixture between a biopolymeric matrix reinforced with a natural material [22,23,24,25]; one of the reinforcers reported for starch composites is the cassava [26].

Garlic waste materials, such as husk, peel, stem, or straw, are materials that do not have an industrial application, despite having interesting properties. Some works report the use of garlic husk (GH) as a source of phenolic compounds [27], for edible films [28,29,30,31,32], and nanocellulose extraction [33], which are some of the explored options for this waste material. GH is one of the garlic waste materials that is a promising additive for polymer matrices. There are various investigations of materials reinforced with natural materials that evaluate their characteristics and potential as alternatives to the use of synthetic materials. A few publications refer to GH used as filler for a polymeric matrix, such as polypropylene [34] or chitosan [35]. The use of GH could provide an advantage due to its chemical composition, in which some phenolic compounds, mainly acids, were recognized, i.e., caffeic, hydroxybenzoic, ferulic, p-coumaric, and chlorogenic acids [36].

Thus, this research aims to obtain a new environmentally friendly product from corn starch reinforced with particles of a GH residue using the evaporative casting method. In addition, the impact of waste, such as GH, and subsequently the structural, thermal, mechanical, and thermo-mechanical properties will be evaluated.

## 2. Materials and Methods

### 2.1. Materials

Cornstarch was purchased from Maizena (Unilever Manufacturera, Tultitlan, Mexico). Glycerin was acquired from La Corona industry (Parque Industrial Xalostoc, Ecatepec, Mexico), and garlic husks (*Allium sativum*) were obtained from the waste of the production of homemade food.

### 2.2. Obtaining Reinforcer

The material used as reinforcer was obtained from *Allium sativum*. Garlic is used in the preparation of most Mexican foods. GH, which is a domestic waste product, has no application in Mexican cuisine, and is therefore thrown away. GH was crushed in an electronic mill and sieved through a 60-mesh screen to obtain particles under 250 µm.

### 2.3. Composites Preparation

The composites were prepared using the evaporative casting technique. A corn starch solution was made with 2 g of starch, 50 mL of distilled water, and 1 mL of glycerin. GH particles were added at 2–10% (*w*/*w*). The solution was heated at 90 ± 5 °C for 10 min with mechanical stirring [12]. The solution was then poured into silicone molds and allowed to cool for 24 h at 30 °C. Table 1 shows the corresponding concentrations and nomenclature used in the synthesis and characterization of composites. 

### 2.4. Composites Characterization

Fourier transform infrared (FTIR) spectra of the films were recorded using an attenuated total refraction (ATR) accessory with a ZnSe plate. Measurements used an FTIR spectrometer (Bruker, Tensor 37, Karlsruhe, Germany) with spectral resolution of 1 cm^−1^ and 32 scans in the wavelength range of 4000–400 cm^−1^. Thermogravimetric analysis (TGA) was carried out with a TA Instrument Q600 analyzer (New Castle, DE, USA) under a nitrogen atmosphere in a temperature range of 30–600 °C with a heating rate of 10 °C/min and using platinum pans. Dynamic mechanical analysis (DMA) was performed on a TA Instruments DMA Q800 analyzer (New Castle, DE, USA) in the multifrequency mode using a dual cantilever clamp at 1 Hz in frequency. The samples were tested from –50 to 100 °C with a heating rate of 5 °C/min. The morphology of the composites was observed by scanning electron microscopy (SEM) using a Hitachi TM-1000 microscope (Tokyo, Japan) at an accelerating voltage of 15 kV. Samples were mounted on metal stubs and were vacuum coated with gold at 7 × 10^−2^ mbar using argon in an EMS 550 sputter coater. The tensile tests were carried out on a mechanical Zwick/Roell model Z005 tester (Ulm, Germany), with a load cell of 5000 N and at a speed of 50 mm/min. Dog-bone shaped specimens were made according to ASTM D638 [37], and five specimens were performed.

## 3. Results

### 3.1. Garlic Husk(GH) Dispersion and Physical Appearance of Composites

**Figure 1:**
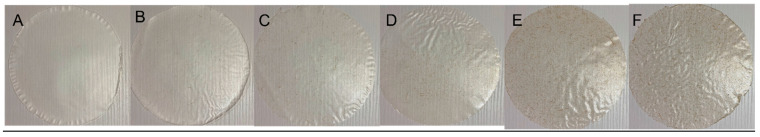
Physical appearance of starch–garlic husk composites. (**A**) Starch, (**B**) SG02, (**C**) SG04, (**D**) SG06, (**E**) SG08, and (**F**) SG10.

Figure 1 shows the starch and the starch–GH composites. The composites show a gradual color change associated with the percentage of added particles. In addition, they do not present saturations or agglomerations when GH particle content increases. This is apparent by the homogeneous distribution, as has been presented in other investigations in which natural fibers have been used [12,38,39]. In addition, adding particles up to 10 wt % allows for good embedding.

### 3.2. Morphology of Composites Studied by SEM

**Figure 2:**
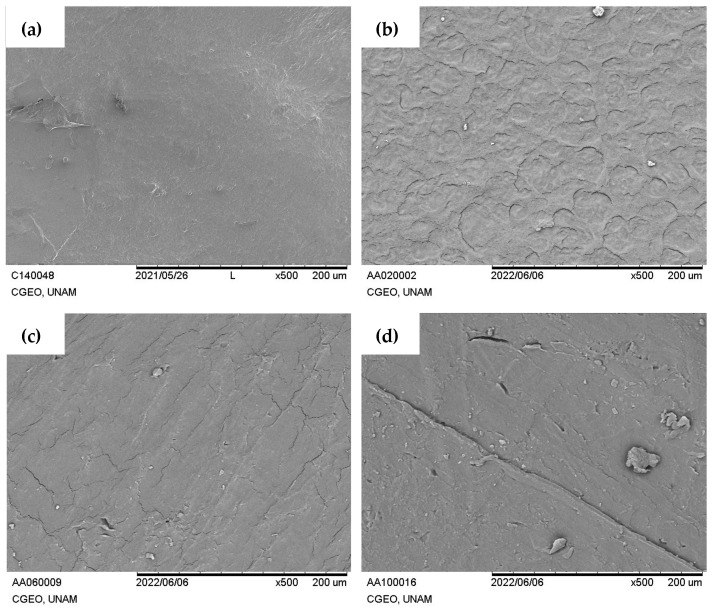
SEM micrographs of starch–garlic husk composites. (**a**) Starch, (**b**) SG02, (**c**) SG06, and (**d**) SG10.

Figure 2a shows the surface of the starch film, in which a smooth surface is observed. Figure 2b–d show the starch–GH composites. In the images, you can see the absence of separation of the composite, where the reinforcement is completely wet by the matrix, which indicates that the films have a good interaction and compatibility, as has been reported in other investigations where starch has been reinforced with hibiscus or keratin fibers [12,38].

The images corroborate the results of the physical appearance presented in Figure 1. Therefore, it is attributed that there are no agglomerations related to the amount of reinforcer used because there are no areas with saturations, as have been presented in other investigations, where the reinforcer was not well dispersed and had areas with a high amount of fiber [38,39].

### 3.3. FTIR Analysis

**Figure 3:**
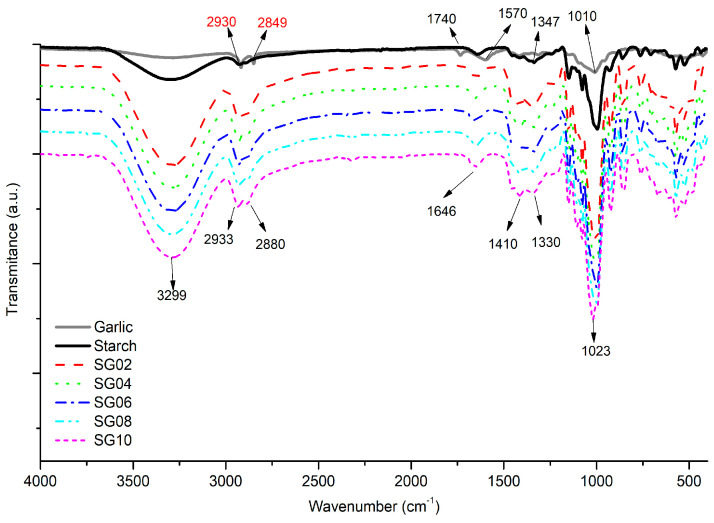
FTIR spectra for starch–garlic husk composites, with 2–10 wt% of particles.

The structural changes in starch biobased films were recorded by FTIR analysis, where pure starch, GH, and starch–GH composites were analyzed. Figure 3 shows the spectra obtained from the pure components and the composite films at different concentrations of GH ranging from 2–10% (*w*/*w*). Pure starch displayed the following main peaks: a fingerprint broadband centered at 3299 cm^−1^ (-OH) of polysaccharides, a 2930 cm^−1^ peak attributed to stretching, as well as a 1330 cm^−1^ bending peak belonging to alkane bands (C-H), and a 1646 cm^−1^ (CH_2_-OH) stretching vibration which only appears in pure starch, all of which are associated with cross-linking of water and starch [40]. Peaks at the 1150 and 1010 cm^−1^ stretching vibrations are related to the C-O-C glucose monomer of starch [41,42]. In the case of pure GH, the signals correspond to cellulose, hemicellulose, and pectin polysaccharides, as well as lignin compounds. Bands at 2930 cm^−1^ and 2849 cm^−1^ are assigned to symmetric and asymmetric alkyl (CH) stretching bands, respectively. Other frequencies include a 1740 cm^−1^ (C=O) stretching vibration from polysaccharides and cellulose compounds, 1603 cm^−1^ (C=C) alkene double bonds (conjugated), and a weak signal at 1570 cm^−1^ that is attributed to the C-N stretching vibration of the amide II peptide group. A 1010 cm^−1^ (C-O-H) peak is associated with the carbohydrate groups present in GH [43].

When starch matrix and GH reinforcer are blended through the casting technique, the linking interactions between them cause shifts and changes in their individual bands. Composite films present an intense band at 3299 cm^−1^ due to OH stretching vibrations from the polysaccharide starch matrix and GH (derived from plants as proteins and carbohydrates) due to the linking process among these components [43]. A proportional increase in the CH_2_ stretching band was observed at 2930 cm^−1^; nevertheless, at 2846 cm^−1^ (C-H), the alkane asymmetric group shifted to 2880 cm^−1^ due to a high sensitivity of this group to establish a linkage with the starch matrix. The pronounced band intensity is correlated to the highest concentration of GH at 10%. The signal at 1646 cm^−1^ assigned to the -OH molecule flexion is associated with the water hygroscopic state of starch. An important linking point between matrix and GH is observed when the band at 1570 cm^−1^ (GH) is combined with the band at 1646 cm^−1^ (starch), finally presenting a single band at 1637 cm^−1^ from aliphatic and aromatic double bonds as well as alcohol groups [44]. This corroborates that starch is working as a matrix in the composite. The combination of bands at 1410 cm^−1^ and 1330 cm^−1^ (C-OH) corresponds to an alcohol deformation vibration of the glycoside monomer. But, it is important to highlight here that this group comes from a starch alcohol, where a peak is first observed at 1347 cm^−1^ (-CH_2_-OH) on the pure starch spectra. Nevertheless, when linkage occurs with GH reinforcer, hydroxyl bonding takes place [43,44]. The increasing intensity of the band at 1023 cm^−1^ is related to a (C-C-O) alcohol asymmetric stretching vibration of the glucose unit group and is the result of the union of the glycosidic groups of starch and GH, confirming that the specific monomer is an active site. The intensity of the band is directly proportional to the increase in the weight percent of the included particles. Meanwhile, the symmetric stretch peak at 858 cm^−1^ (C-O-C) is attributed to ether linkage from the starch matrix [43]. Finally, the signals around 858–764 cm^−1^ can be related to S-S and/or C-S polysulfide groups, which also participate in the bonding process among starch matrix and garlic husk reinforcement [43].

### 3.4. TGA Analysis

**Figure 4:**
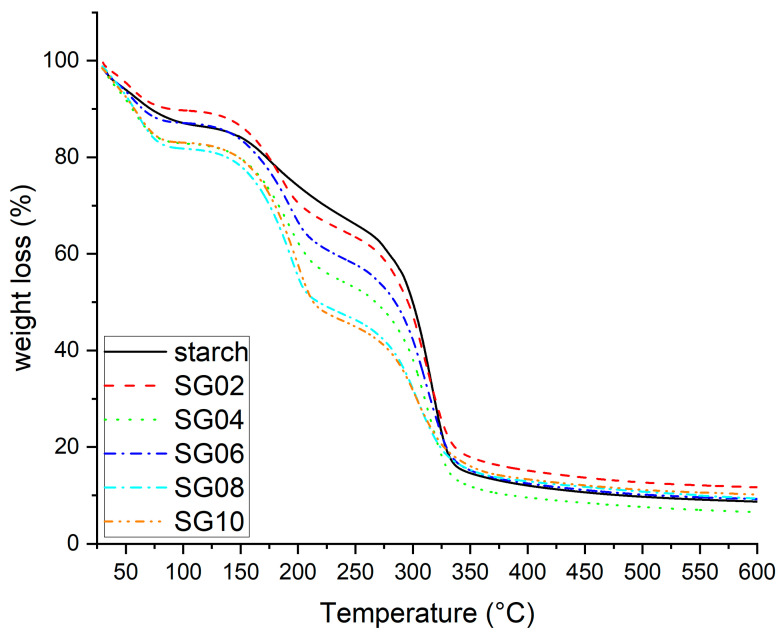
TGA thermograms for starch–garlic husk composites, with 2–10 wt % of particles.

The TGA technique is commonly used to analyze the thermal stability and degradation of materials. When GH particles are added to starch biocomposites, they may exhibit different thermal properties than pure starch due to the presence of constituents of GH particles. Incorporating GH into the starch polymers leads to a change in the TGA thermogram, which is observed in Figure 4. The TGA thermograms of the composite films demonstrate a gradual weight loss around 80–130 °C due to the vaporization of physically and chemically bound water, with a weight loss of approximately 15% wt in that region [45]. The second weight loss range, which exhibits a loss between 210 and 350 °C, corresponds to the structural degradation of the starch films/GH particles due to the pyrolysis of starch and cellulose present in the biocomposite, resulting in a weight loss of about 70% wt, which is the highest weight loss stage for the composites film. The weight loss above 350 °C could be attributed to the decomposition of the remaining organic matter [46]. Total weight loss was >90% wt at 600 °C. In summary, films reinforced with garlic husk particles exhibit a lower thermal stability compared to pure starch only; the SG02 is evidenced in the light increase in decomposition temperature in the analysis. Additionally, the amount of carbon residue in each composite is influenced by the GH particles.

**Figure 5:**
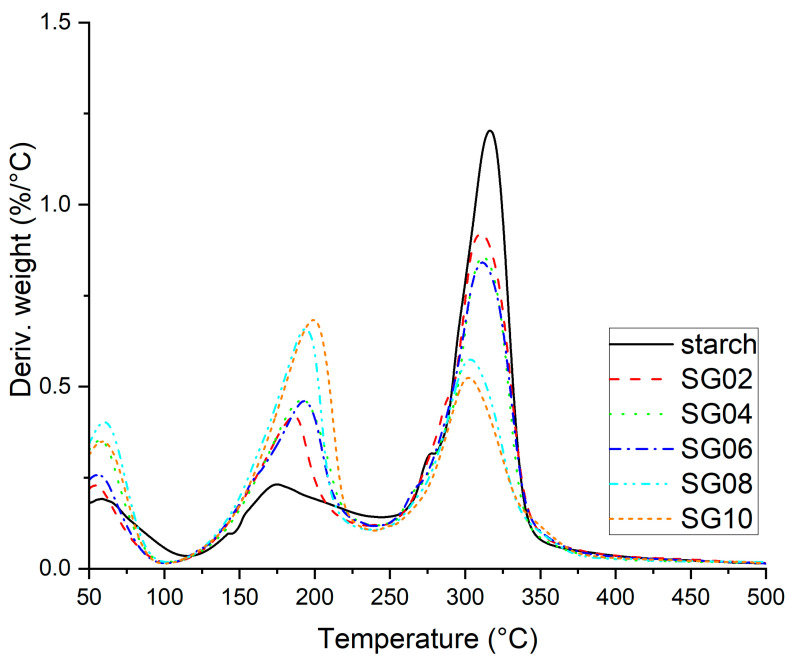
DTG thermograms for starch–GH composites, with 2–10 wt % of particles.

The DTG peak represents the medium decomposition temperature and helps to identify how quickly a material decomposes. Figure 5 depicts the weight loss derivative (DTG) curve of TGA thermogram, and it is possible to identify the three decomposition peaks, similar to those previously described, the first between 50 and 100 °C, which is due the dehydration of films, and the second peak between 160 and 250 °C, which indicates the decomposition of polysaccharides present in starch and GH [34,47]. It is evident that this peak is lower for starch and it increases when GH is added; this can be associated with the composition of GH which includes pectin, hemicellulose, and other polysaccharides, as previously reported [34]. The third peak (310 °C) is associated with the decomposition and depolymerization of cellulose, with lignin being present in GH and other organic components being present in starch. Another interesting observation is that peaks may vary according to the GH content, which indicates that susceptibility to the decomposition may vary. When the peak increases, this indicates that the material lost more weight. In this case, the peak at 310 °C decreases when GH increases in the formulation in comparison with starch; therefore, these components are less susceptible to decompose.

### 3.5. DMA Analysis

**Figure 6:**
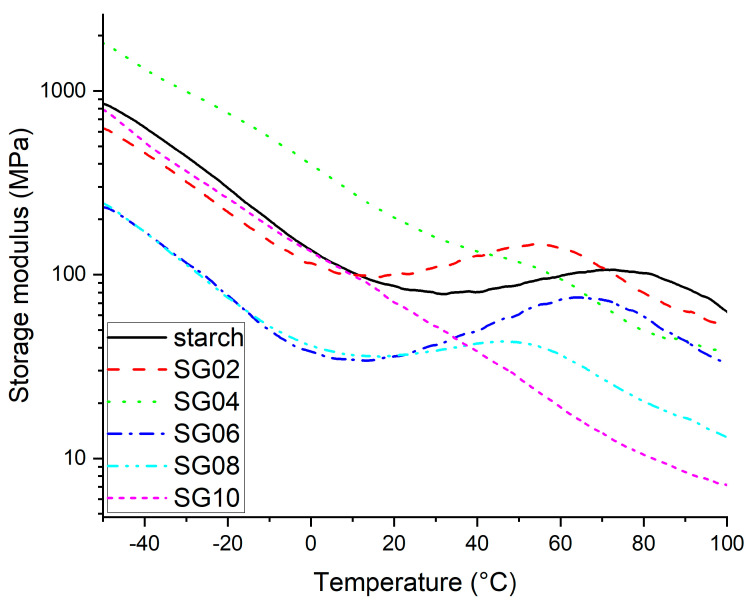
DMA thermogram of storage modulus starch–GH composites, with 2–10 wt % of particles.

Figure 6 shows the DMA thermogram for starch and GH composites, and it is possible to observe the effect of addition of GH reinforcement. In this figure, it is possible to observe that the typical dynamic mechanical behavior of storage modulus for starch can be seen under a temperature range, and it is evident that the stiffness decreases as a function of the temperature. In addition, it is regarded as a material ability to store applied energy for future purposes, which provides information about the stiffness of the material under cyclic stress [46,48,49,50,51]. Usually, the addition of a reinforcer material in a polymer matrix improves its mechanical properties because the applied stress can be transferred to the reinforcement, which helps the polymer matrix during the mechanical stress and associates the filler dispersion and compatibility between the materials to be mixed.

According to Figure 6, the SG04 composite is the only one that shows an improvement over the evaluated test range temperature in comparison with the starch film. The rest of the composites show an interesting behavior of increasing the storage modulus around 20 °C, and then slightly diminishing. It is obvious that only the SG04 showed an increase of 115% compared with the pure starch at −50 °C, indicating that at a lower percentage of reinforcement, the dispersion of the GH into the starch matrix is desired. This behavior remains even at room temperature (25 °C), showing an increase compared to the raw starch. Both observations are very relevant because this composite presents higher stiffness in the temperature, which is usually employed by packaging-type bags for foods (sub-ambient and room temperature). This behavior is also observed for starch and composites SG02, SG06, and SG08. Additionally, it is attributed to a plasticized effect and a possible evaporation of water, which is due the hydrophilic nature of the polysaccharides that reflects an absorption of water [52]. Also, at higher particles content, it is possible that the agglomeration generates poor stress distribution and a decrease in stiffness [53].

Although there are no reports on the use of GH as reinforcement in starch, there is a work that reports the use of GH as reinforcement in polypropylene (PP); in this study, they found an increase in the storage modulus by including GH in a PP matrix, even at high reinforcement percentages at sub-ambient temperature [34] or in biopolymers as the chitosan, where the results showed good compatibility. These results were attributed to the presence of phenolic compounds in the GH, with OH groups forming a hydrogen bond with the pure chitosan [35]. In the same way, in another study, starch was reinforced with cellulose obtained from garlic stalks. In all samples, the modulus was higher than the raw starch which is attributed to the presence of hydroxyl groups of the cellulose and in the starch, promoting the formation of hydrogen bonds [45].

**Figure 7:**
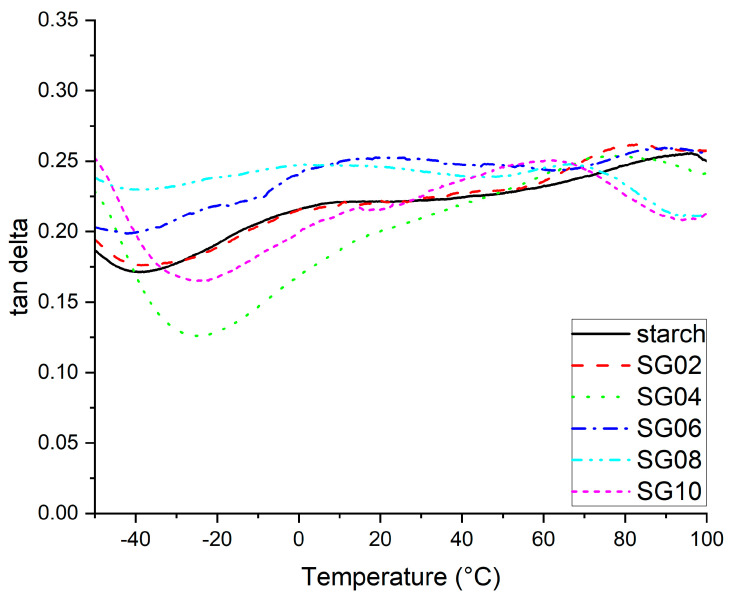
DMA thermogram of Tan δ starch–GH composites, with 2–10 wt % of particles.

In Figure 7, Tan δ curve from DMA is plotted, which represents the relationship between the loss and storage modulus. A high Tan δ value indicates a material with high, non-elastic strain component and a low value indicates high elasticity. Also, this signal can be used to evaluate the interaction level between the matrix and reinforcer [54]. The onset of this curve shows a shift of the transition observed around −50 °C, which is attributed to the glycerol-rich-phase (β relaxation) [55,56,57]. As can be seen, the curve of SG04 and SG10 evidenced a shift in the valley at −15 °C. From these results, a displacement in the peaks of the plasticizer-rich phase can be inferred from the GH interaction, which reflects $\left(T\beta \right)$ [58]. Another signal is observed between −5 and 20 °C, which can be attributed to the α relaxation due to the altered chains due to the presence of GH; this signal appears clearly for SG06 and SG08, while for the other composites, it can just be observed as a shoulder. The Tan δ of SG06 and SG08 confirms that the higher percentages of added GH evidenced poor mechanical properties, as noted in the storage modulus and tensile test. The glass transition for starch–GH composites appeared from 50 to 90 °C, which is consistent as reported by other authors [51,52]. Starch evidenced a peak at approximately 90 °C, then a reduction in Tan δ. As reported by Pavon et al. [55], a reduction in the T_g_ can be related to an increment in chain mobility due to the plasticizer effect [59,60]; in the case of SG02, there is a positive effect as the storage modulus and tensile response are evidenced. However, as the percentage of GH increases, the plasticizer effect changes to a negative effect on the performance of composites due to possible particle agglomerations. Also, the high polarity of starch is diminished by the GH, which allows for the high motion chains [49].

### 3.6. Tensile Tests

GH waste constitutes around a fifth part of the total garlic production in the world, resulting in approximately 3 million tons of waste per year [61]. It is chemically composed of 41–45% cellulose, 7.6–17.5% hemicellulose, and 25–34% lignin, among other components [62]. This high content cellulose material could be used as an additive in biodegradable composites, avoiding its final disposal [52]. Starch is an abundant natural polymer; however, it tends to have poor mechanical properties [63]. As other authors have reported [64], cellulose-based materials with phenolic compounds such as GH can be used to improve the mechanical properties of starch [65,66].

**Figure 8:**
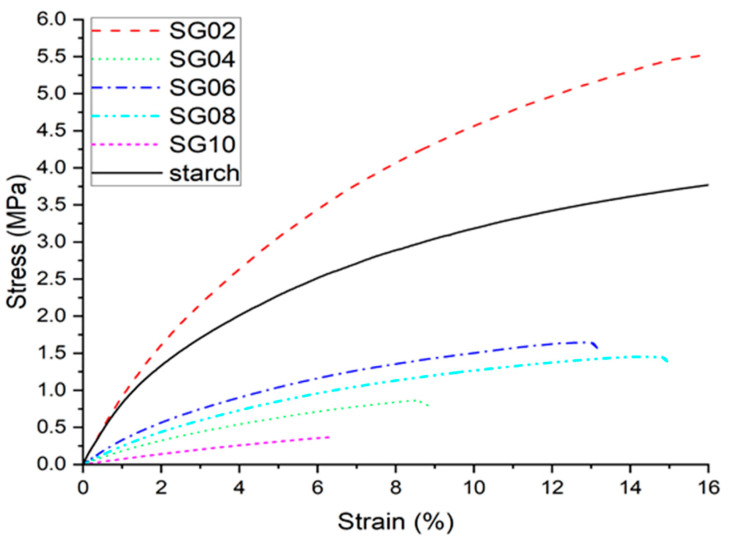
Stress–strain curves for starch–GH composites, with 2–10 wt % of particles.

Stress–strain curves for both starch and composites are shown in Figure 8. Young’s modulus and the tensile strength of composite films were measured. Also, Table 2 summarizes the values obtained from Figure 7. As can be seen, the incorporation of GH influences most of the composite’s properties to lower values for Young’s modulus, tensile strength, and the calculated area. Chaudhary et al. (2021) [35] reported that chitosan films with similar amounts of GH increased their mechanical properties due to interactions with phenolic compounds. However, the SG02 sample exhibited higher modulus, around 11% more than starch. As was observed in the physical appearance of films, the GH was better dispersed in the matrix. This could be the reason for the higher stress transmission from the matrix to the cellulose. In addition, the amylose and amylopectin -OH groups could form hydrogen bonds with phenolic compounds in GH [23,35]. Then, after the addition of 4 wt % of GH, the mechanical properties decreased as shown in Figure 7. Zhang et al. (2023) mentioned that phenolic compounds could bind to starch; thus, in the presence of rich hydroxyl content material, starch could aggregate due to the alteration of its microstructure [23]. Liu et al. (2020) reported an experiment during the gelatinization of starch, and they mentioned that hydrogen bonds and Van der Walls forces tend to form intermolecular aggregates in starch composites. Also, the molecular size, number, and distribution influence the intermolecular interactions from the materials added to starch [67].

## 4. Conclusions

In this work, composite materials were obtained from the combination of starch and GH particles. GH, considered as waste, was used as a reinforcing material. Films were obtained using an evaporative casting technique. The morphology of the films through images and micrographs showed a good distribution of the reinforcer particles. The increase in reinforcer particles did not result in agglomerations and the adhesion and interaction between both materials were confirmed. The structural changes in the films were identified through FTIR spectra, in which the interaction and formation of bonds were verified. These interactions caused displacements and changes in individual bands due to the high sensitivity of the reinforcer group to establish bonds with the starch matrix. TGA showed that the films reinforced with the minimum concentration of GH particles showed a slight increase in decomposition temperature in the analysis compared to pure starch. On the other hand, according to DMA, the composite SG04 showed an increase of 115% compared to pure starch and an interesting behavior at low temperatures, which can be a possibility for packing food applications. The mechanical tests of the composites showed a less rigid behavior than the starch matrix. Finally, the composites obtained could be an environmental alternative using non-synthetic materials from waste, and contribute to a circular economy. Therefore, research into this type of material would offer an alternative to materials science and industry, avoiding an increase in carbon footprint due to the use of single-use and petroleum-based plastics.

## Figures and Tables

**Table 1:** Composition and nomenclature of starch–garlic husk fiber composites.

Percentage of GH Particles (% wt)	Composites Code
0	starch
2	SG02
4	SG04
6	SG06
8	SG08
10	SG10

**Table 2:** Mechanical properties of starch–GH composites.

Sample	Young’s Modulus (MPa)	Yield Stress (MPa)	Tensile Stress at Break (MPa)	% Elongation at Break
Starch	82.33 ± 10.40	1.04 ± 0.12	4.09 ± 0.23	22.87 ± 0.13
SG02	91.49 ± 4.90	1.53 ± 0.08	5.58 ± 0.25	17.25 ± 0.14
SG04	19.6 ± 2.48	0.32 ± 0.03	0.86 ± 0.14	8.86 ± 0.04
SG06	35.84 ± 0.99	0.54 ± 0.00	1.64 ± 0.03	13.15 ± 0.09
SG08	26.23 ± 2.70	0.44 ± 0.04	1.45 ± 0.13	14.94 ± 0.06

## Data Availability

Data are contained within the article.

## References

[1] de Las Heras R.B., Ayala S.F., Salazar E.M., Carrillo F., Cañavate J., Colom X. (2023). Circular Economy Insights on the Suitability of New Tri-Layer Compostable Packaging Films after Degradation in Storage Conditions. Polymers.

[2] Almeida S., Ozkan S., Gonçalves D., Paulo I., Queirós C.S., Ferreira O., Bordado J., Galhano dos Santos R. (2023). A Brief Evaluation of Antioxidants, Antistatics, and Plasticizers Additives from Natural Sources for Polymers Formulation. Polymers.

[3] Muñoz-Gimena P.F., Oliver-Cuenca V., Peponi L., López D. (2023). A review on reinforcements and additives in starch-based composites for food packaging. Polymers.

[4] Cui C., Zhao S., Zhang Z., Li M., Shi R., Sun Q. (2023). Preparation, and characterization of corn starch straws with strong mechanical properties by extrusion and retrogradation. Ind. Crops Prod..

[5] Melo P.M.A., Macêdo O.B., Barbosa G.P., Santos A.S.F., Silva L.B. (2021). Reuse of natural waste to improve the thermal stability, stiffness, and toughness of postconsumer polypropylene composites. J. Polym. Environ..

[6] Teacă C.A., Shahzad A., Duceac I.A., Tanasă F. (2023). The Re-/Up-Cycling of Wood Waste in Wood–Polymer Composites (WPCs) for Common Applications. Polymers.

[7] Brunšek R., Kopitar D., Schwarz I., Marasović P. (2023). Biodegradation Properties of Cellulose Fibers and PLA Biopolymer. Polymers.

[8] Wandosell G., Parra-Meroño M.C., Alcayde A., Baños R. (2021). Green Packaging from Consumer and Business Perspectives. Sustainability.

[9] Zhu Z., Liu W., Ye S., Batista L. (2022). Packaging design for the circular economy: A systematic review. Sustain. Prod. Consum..

[10] Li Z., Guan J., Yan C., Chen N., Wang C., Liu T., Cheng F., Guo Q., Zhang X., Ye X. (2023). Corn straw core/cellulose nanofibers composite for food packaging: Improved mechanical, bacteria blocking, ultraviolet and water vapor barrier properties. Food Hydrocoll..

[11] Flores-Hernández C.G., Cornejo-Villegas M.D.L.A., Moreno-Martell A., Del Real A. (2021). Synthesis of a biodegradable polymer of poly (sodium alginate/ethyl acrylate). Polymers.

[12] Ortuño-López M.B., Salazar-Cruz B.A., del Real A., Almendarez-Camarillo A., López-Barroso J., Rivera-Armenta J.L., Flores-Hernández C.G. (2023). Physical properties of thermoplastic cornstarch/Hibiscus sabdariffa fiber obtained by evaporation casting. Starch-Stärke.

[13] Hernández-Varela J.D., Chanona-Pérez J.J., Resendis-Hernández P., Victoriano L.G., Méndez-Méndez J.V., Cárdenas-Pérez S., Benavides H.C. (2022). Development, and characterization of biopolymers films mechanically reinforced with garlic skin waste for fabrication of compostable dishes. Food Hydrocoll..

[14] Castro-Yobal M.A., Contreras-Oliva A., Saucedo-Rivalcoba V., Rivera-Armenta J.L., Hernández-Ramírez G., Salinas-Ruiz J., Herrera-Corredor A. (2021). Evaluation of physicochemical properties of film-based alginate for food packing applications. e-Polymers.

[15] Koo S.H., Lee K.Y., Lee H.G. (2021). Effect of cross-linking on the physicochemical and physiological properties of corn starch. Food Hydrocoll..

[16] Nordin N., Othman S.H., Rashid S.A., Basha R.K. (2020). Effects of glycerol and thymol on physical, mechanical, and thermal properties of corn starch films. Food Hydrocoll..

[17] Wang B., Gao W., Kang X., Dong Y., Liu P., Yan S., Yu B., Guo L., Abd El-Aty A.M. (2021). Structural changes in corn starch granules treated at different temperatures. Food Hydrocoll..

[18] Fabra M.J., López-Rubio A., Ambrosio-Martín J., Lagaron J.M. (2016). Improving the barrier properties of thermoplastic corn starch-based films containing bacterial cellulose nanowhiskers by means of PHA electrospun coatings of interest in food packaging. Food Hydrocoll..

[19] Wang S., Zhang P., Li Y., Li J., Li X., Yang J., Ji M., Li F., Zhang C. (2023). Recent Advances and Future Challenges of the Starch-Based Bio-Composites for Engineering Applications. Carbohydr. Polym..

[20] Pradeep M., Binoy R.F., Yaswanth S., Pullan T.T., Joseph M. (2022). Investigations on chitin and coconut fiber reinforcements on mechanical and moisture absorption properties of corn starch bioplastics. Mater. Today Proc..

[21] Cruz-Balaz M.I., Bósquez-Cáceres M.F., Delgado A.D., Arjona N., Morera Córdova V., Álvarez-Contreras L., Tafur J.P. (2023). Green Energy Storage: Chitosan-Avocado Starch Hydrogels for a Novel Generation of Zinc Battery Electrolytes. Polymers.

[22] Othman S.H., Nordin N., Azman N.A.A., Tawakkal I.S.M.A., Basha R.K. (2021). Effects of nanocellulose fiber and thymol on mechanical, thermal, and barrier properties of corn starch films. Int. J. Biol. Macromol..

[23] Zhang W., Roy S., Ezati P., Yang D.P., Rhim J.W. (2023). Tannic acid: A green crosslinker for biopolymer-based food packaging films. Trends Food Sci. Technol..

[24] Chhatariya H.F., Srinivasan S., Choudhary P.M., Begum S.S. (2022). Corn starch biofilm reinforced with orange peel powder: Characterization of physicochemical and mechanical properties. Mater. Today Proc..

[25] Naidu M., Bhosale A., Munde Y., Salunkhe S., Hussein H.M.A. (2022). Wear and Friction Analysis of Brake Pad Material Using Natural Hemp Fibers. Polymers.

[26] Versino F., Lopez O.V., Garcia M.A., Zaritzky N.E. (2016). Starch-based films and food coatings: An overview. Starch-Stärke.

[27] Kallel F., Driss D., Chaari F., Belghith L., Bouaziz F., Ghorbel R., Chaabouni S.E. (2014). Garlic (*Allium sativum* L.) husk waste as a potential source of phenolic compounds: Influence of extracting solvents on its antimicrobial and antioxidant properties. Ind. Crops Prod..

[28] Roy S., Das T., Goh K.L., Verma C., Maji P.K., Sharma K., Chang Y.W. (2022). Synergistic effects of fresh garlic juice in cellulose based antimicrobial food packaging film. Mater. Lett..

[29] Ding Y., Jiang Y., Zhong Y., Wang D., Deng Y., Meng F., Li Y., Zhang M., Zhang C. (2023). Preparation of garlic stem cellulose nanocrystal/leaf extract/chitosan film for black garlic preservation by electrostatic spraying. Int. J. Biol. Macromol..

[30] Abid M.A., Abid D.A., Aziz W.J., Rashid T.M. (2021). Iron oxide nanoparticles synthesized using garlic and onion peel extracts rapidly degrade methylene blue dye. Phys. B.

[31] Kumar A., Patel G., Dwivedi M., Hashmi S., Pradhan R.C. (2022). Synthesis and Characterization of Edible Films from Garlic (*Allium sativum*) Husk Components. J. Sci. Ind. Res..

[32] Senthilkumar A., Muthuswamy R., Maheshwari-Nallal U., Ramaiyan S., Kannan P., Muthupandi S., Lakshminarayanan S.P., Sambasivm S., Ayyar M. (2023). Green Synthesis of copper nanoparticles from agro-waste garlic husk. Int. J. Chem. Phys..

[33] Gao X., Jia Y., Chen Z., Santhanam R.K., Zhang M., He C., Chen H. (2022). Synthesis of hydrogels based on nanocellulose from garlic straw and regulating the release of allicin and its cytotoxicity. Food Sci. Tech..

[34] Villalobos-Neri E.E., Macchlesh Del Pino-Perez L.A., Velasco-Ocejo H.A., Rivera-Armenta J.L., Espindola-Flores A.C. (2023). Preparation and Evaluation of Thermal Properties of Composites Based on Polypropylene Reinforced with Garlic Husk Particles (GHP). J. Nat. Fibers.

[35] Chaudhary B.U., Lingayat S., Banerjee A.N., Kale R.D. (2021). Development of multifunctional food packaging films based on waste Garlic peel extract and Chitosan. Int. J. Biol. Macromol..

[36] Kopec A., Skoczylas J., Jedrszczyk E., Francik R., Bystrowska B., Zawistowski J. (2020). Chemical Composition and Concentration of Bioactive Compounds in Garlic Cultivated from Air Bulbils. Agriculture.

[37] (2010). Standard Test Method for Tensile Properties of Plastics.

[38] Flores-Hernandez C.G., Colin-Cruz A., Velasco-Santos C., Castano V.M., Rivera-Armenta J.L., Almendarez-Camarillo A., Garcia-Casillas P.E., Martinez-Hernandez A.L. (2014). All Green Composites from Fully Renewable Biopolymers: Chitosan-Starch Reinforced with Keratin from Feathers. Polymers.

[39] Flores-Hernandez C.G., Colin-Cruz A., Velasco-Santos C., Castano V.M., Almendarez-Camarillo A., Olivas-Armendariz I., Martinez-Hernandez A.L. (2018). Chitosan–Starch–Keratin Composites: Improving ThermoMechanical and Degradation Properties Through ChemicalModifiation. J. Polym. Environ..

[40] Patil S., Bharimalla A.K., Mahapatra A., Dhakane-Lad J., Arputharaj A., Kumar M., Raja A.S.M., Kambli N. (2021). Effect of polymer blending on mechanical and barrier properties of starch-polyvinyl alcohol based biodegradable composite films. Food Biosci..

[41] Xiong H., Tang S., Tang H., Zou P. (2008). The structure and properties of a starch-based biodegradable film. Carbohydr. Polym..

[42] Sarkar A., Biswas D.R., Datta S.C., Dwivedi B.S., Bhattacharyya R., Kumar R., Bandyopadhyay K.K., Saha MChawla G., Saha J.K., Patra A.K. (2021). Preparation of novel biodegradable starch/poly (vinyl alcohol)/bentonite grafted polymeric films for fertilizer encapsulation. Carbohydr. Polym..

[43] Biancolillo A., Marini F., D’Archivio A.A. (2020). Geographical discrimination of red garlic (*Allium sativum* L.) using fast and non-invasive Attenuated Total Reflectance-Fourier Transformed Infrared (ATR-FTIR) spectroscopy combined with chemometrics. J. Food Compos. Anal..

[44] Dharmalingam K., Ramachandran K., Sivagurunathan P. (2007). FTIR and dielectric studies of molecular interaction between alkyl methacrylates and primary alcohols. Phys. B.

[45] Agustin M.B., Ahmmad B., De Leon E.R.P., Buenaobra J.L., Salazar J.R., Hirose F. (2013). Starch-based biocomposite films reinforced with cellulose nanocrystals from garlic stalks. Polym. Compos..

[46] Lee S.Y., Mohan D.J., Kang I.A., Doh G.H., Lee S., Han S.O. (2009). Nanocellulose reinforced PVA composite films: Effects of acid treatment and filler loading. Fibers Polym..

[47] Reddy J.P., Rhim J.W. (2014). Isolation and characterization of cellulose nanocrystals from garlic skin. Mater. Lett..

[48] Zhang Y., Rempel C., Liu Q. (2014). Thermoplastic starch processing and characteristics—A review. Crit. Rev. Food Sci. Nutr..

[49] Garcia P.S., Turbiani F.R.B., Baron A.M., Brizola G.L., Tavares M.A., Yamashita F., Eiras D., Grossmann M.V.E. (2018). Sericin as compatibilizer in starch/polyester blown fims. Polimeros.

[50] Ghizdareanu A.I., Banu A., Pasarin D., Ionita A., Nicolae C.A., Gabor A.R., Pătroi D. (2023). Enhancing the Mechanical Properties of Corn Starch Films for Sustainable Food Packaging by Optimizing Enzymatic Hydrolysis. Polymers.

[51] Mucha M., Pawlak A. (2005). Thermal analysis of chitosan and its blends. Thermochim. Acta.

[52] Salim M.H., Kassab Z., Abdellaoui Y., García-Cruz A., Soumare A., Ablouh E.H., El Achaby M. (2022). Exploration of multifunctional properties of garlic skin derived cellulose nanocrystals and extracts incorporated chitosan biocomposite films for active packaging application. Int. J. Biol. Macromol..

[53] Saba N., Jawaid M., Alothman O.Y., Paridah M.T. (2016). A review on dynamic mechanical properties of natural fibre reinforced polymer composites. Constr. Build. Mater..

[54] Martone A., Formicola C., Giordano M., Zarrelli M. (2010). Reinforcement efficiency of multi-walled carbon nanotube/epoxy nano composites. Compos. Sci. Tech..

[55] Pavon C., Aldas M., López-Martínez J., Hernández-Fernández J., Arrieta M.P. (2021). Films based on thermoplastic starch blended with pine resin derivatives for food packaging. Foods.

[56] Castro J.M., Montalbán M.G., Martínez-Pérez N., Domene-López D., Pérez J.M., Arrabal-Campos F.M., Fernández I., Martín-Gullón I., García-Quesada J.C. (2023). Thermoplastic starch/polyvinyl alcohol blends modification by citric acid–glycerol polyesters. Int. J. Biol. Macromol..

[57] Torres F.G., Mayorga J.P., Vilca C., Arroyo J., Castro P., Rodriguez L. (2019). Preparation, and characterization of a novel starch–chestnut husk biocomposite. SN Appl. Sci..

[58] Taguet A., Huneault M.A., Favis B.D. (2009). Interface/morphology relationships in polymer blends with thermoplastic starch. Polymer.

[59] DeFelice J., Lipson J. (2021). The Influence of Additives on Polymer Matrix Mobility and the Glass Transition. Soft Matter.

[60] Liu P., Yu L., Wang X., Li D., Chen L., Li X. (2010). Glass transition temperature of starches with different amylose/amylopectin ratios. J. Cereal Sci..

[61] Raaj E.P., Bhuvaneshwari K., Lakshmipathy R., Devi V.V., Rico I.L.R. (2022). Garlic peel surface modification and fixed-bed column investigations towards crystal violet dye. Adsorpt. Sci. Tech..

[62] Hernández-Varela J.D., Chanona-Pérez J.J., Benavides H.A.C., Sodi F.C., Vicente-Flores M. (2021). Effect of ball milling on cellulose nanoparticles structure obtained from garlic and agave waste. Carbohydr. Polym..

[63] do Lago R.C., de Oliveira A.L.M., dos Santos A.D.A., Zitha E.Z.M., Carvalho E.E.N., Tonoli G.H.D., Boas E.V.D.B.V. (2021). Addition of wheat straw nanofibrils to improve the mechanical and barrier properties of cassava starch–based bionanocomposites. Ind. Crops Prod..

[64] Harussani M.M., Sapuan S.M., Firdaus A.H.M., El-Badry Y.A., Hussein E.E., El-Bahy Z.M. (2021). Determination of the tensile properties and biodegradability of cornstarch-based biopolymers plasticized with sorbitol and glycerol. Polymers.

[65] Lim H., Hoag S.W. (2013). Plasticizer Effects on Physical–Mechanical Properties of Solvent Cast Soluplus^®^ Films. AAPS Pharm. Sci. Tech..

[66] Chen N., Gao H.-X., He Q., Zeng W.-C. (2023). Potential application of phenolic compounds with different structural complexity in maize starch-based film. Food Struct..

[67] Liu B., Zhong F., Yokoyama W., Huang D., Zhu S., Li Y. (2020). Interactions in starch co-gelatinized with phenolic compound systems: Effect of complexity of phenolic compounds and amylose content of starch. Carbohydr. Polym..

